# Same-day discharge after duodenal endoscopic submucosal dissection and hybrid endoscopic submucosal dissection: a case series of 10 patients

**DOI:** 10.1016/j.igie.2026.01.013

**Published:** 2026-02-02

**Authors:** Sayaka Nagao, Makoto Nishimura, Monika Laszkowska, Mako Koseki, Laura Tang, Mark A. Schattner

**Affiliations:** 1 Gastroenterology, Hepatology, and Nutrition Service, Memorial Sloan Kettering Cancer Center, New York, New York, USA; 2 Department of Pathology and Laboratory Medicine, Memorial Sloan Kettering Cancer Center, New York, New York, USA

## Abstract

**Background and Aims:**

Duodenal endoscopic submucosal dissection (ESD) involves postprocedural hospitalization owing to concerns about delayed adverse events. The safety of same-day discharge (SDD) after duodenal ESD remains unexplored. This case series reports clinical outcomes of patients undergoing SDD after duodenal ESD.

**Methods:**

This case series included 10 patients receiving SDD after duodenal ESD at a tertiary referral center.

**Results:**

Ten patients (5 males) with a mean (standard deviation) age of 62.6 (12.4) years were included. Mean resection size and procedure time were 12.5 (4.5) mm and 66.5 (32.3) minutes, respectively. Complete mucosal defect closure was achieved using endoscopic clips in all patients. No intraoperative or delayed adverse events were observed.

**Conclusions:**

SDD appears safe in carefully selected patients with small lesions undergoing duodenal ESD by experienced endoscopists. These findings provide initial evidence supporting the consideration of SDD after duodenal ESD, although larger studies are needed to establish standardized protocols.

## Introduction

Endoscopic submucosal dissection (ESD) is a minimally invasive method for treating superficial duodenal neoplasms,^1^, ^2^, ^3^ offering the potential for en bloc resection and precise histological assessment. However, the thin wall of the duodenum and its complex anatomy make ESD technically challenging compared to that in other parts of the gastrointestinal tract, with relatively high rates of adverse events, including perforation (0%-36%) and bleeding (0%-22%).^4^^,^^5^ These relatively high adverse event rates have traditionally led to postprocedural inpatient observation. However, the reported length of hospitalization varies considerably across health care systems. In Asia, the average hospital stay ranges from 5 to 11 days, with many patients being observed for more than a week.^4^^,^^6^ In contrast, in the United States, shorter hospital stays are reported, with an average length of approximately 1 day, and same-day discharge (SDD) is being implemented in some cases.^3^

Although promising results have been achieved with SDD after ESD in other parts of the gastrointestinal tract,^7^^,^^8^ evidence specific to duodenal ESD remains limited, to our knowledge. Previous studies have focused primarily on esophageal, gastric, and colorectal ESD procedures, leaving duodenal ESD largely unexplored in this context. To address this knowledge gap, we report our experience with SDD after duodenal ESD in a consecutive case series.

## Methods

### Study design and patient population

This case series included 10 patients for whom SDD was implemented after they underwent duodenal ESD or hybrid ESD at the Memorial Sloan Kettering Cancer Center, New York, New York, USA, between October 1, 2018, and November 28, 2023. This study was approved by the Institutional Review Board (protocol approval no. 20-495A). Hybrid ESD is defined as any procedure that incorporates snare resection, even partially, primarily for safety considerations and procedural efficiency in challenging cases. Duodenal ESD was performed for lesions with fibrosis, suspected submucosal invasion, or cases when endoscopic mucosal resection was technically difficult or had previously failed. Ampullary lesions were excluded.

### ESD procedure and postprocedural management

All ESD procedures were performed by a single experienced endoscopist with over 1500 ESD procedures completed across various regions of the gastrointestinal tract. Complete closure of the mucosal defect was achieved using through-the-scope clips in all patients. SDD criteria included residence within a reasonable travel distance, no muscularis propria injury, complete mucosal defect closure, and asymptomatic status during recovery. Some patients were hospitalized based on the endoscopist’s comprehensive assessment, which included considerations such as patient preference and advanced age. Postanesthesia care unit stay was comparable to that following other endoscopic procedures. Postoperative diet progressed from liquids on day 0 to soft food on days 1 and 2, followed by a regular diet. Proton pump inhibitors were administered for at least 4 weeks.

Adverse events included the need for emergency department visit, rehospitalization, and medical or endoscopic intervention for bleeding or perforation. Intraoperative bleeding was defined as requiring difficult endoscopic hemostasis or a blood transfusion. Postoperative bleeding was considered an adverse event if endoscopic treatment or transfusion was required. Procedure time was measured from scope insertion to removal. The size of the resected specimen and resection margins (R0, curative resection with negative resection margins; R1, positive resection margins; Rx, presence of residual tumor cannot be assessed) were recorded.

### Follow-up protocol

All patients received standardized discharge instructions with explicit criteria for immediate contact. Systematic follow-up was conducted with at least 1 scheduled clinical evaluation (in-person, telemedicine, or phone) for all patients. Nine patients continued follow-up at our institution beyond 30 days, whereas 1 patient was transferred to another facility for ongoing care closer to home after 23 days. Electronic medical records were reviewed to assess adverse events occurring within 30 days after the procedure.

### Data collection and outcome measures

Demographic, clinical, and procedural data were collected from electronic medical records. Lesions were categorized according to the Paris classification,^9^ when applicable. Continuous variables are presented as the mean (standard deviation).

## Results

### Patient characteristics

Fifty-two duodenal ESD procedures were performed at our institution between October 1, 2018, and November 28, 2023. Of these patients, 10 (19.2%) met the SDD criteria (Tables 1 and 2). The mean patient age was 62.6 (12.4) years, and 50% of the patients were male. The tumors were predominantly located in the duodenal bulb (50%) and the second portion (30%), and the mean resection size was 12.5 (4.5) mm. Notably, 90% of the patients had American Society of Anesthesiologists physical status III, reflecting the complexity of our referral population. Histologically, 5 (50%) cases were adenomas, and the remaining 5 (50%) were neuroendocrine tumors. In the present cases, 4 patients were taking aspirin at 81 mg. For patients taking aspirin prophylactically without a clear medical indication, the medication was withheld for 5 to 7 days before and after ESD, following consultation with the primary care physician. In cases where aspirin was medically necessary, we consulted the patient’s cardiologist and proceeded with ESD while continuing aspirin, in accordance with American Society for Gastrointestinal Endoscopy guidelines.^10^

**Table 1:** Case details

Case	Sex	Age, years	Location	ASA physical status classification	Macroscopic type	Resection size, mm	Histologic type	En bloc resection	Horizontal margins	Vertical margins	Resection	Total procedure time, minutes	Previous treatment	Procedure type	Closure status (complete/partial)	Number of clips	Initial post-ESD outpatient follow-up, days	Postprocedural adverse events
1	Male	73	Bulb	III	Subepithelial lesion	10	Neuroendocrine tumor	Yes	Negative	Negative	R0	50	No	ESD	Complete	5	8	No
2	Female	59	Bulb	III	Subepithelial lesion	9	Neuroendocrine tumor	Yes	Negative	Positive	R1	33	No	Hybrid ESD	Complete	5	16	No
3	Female	71	Third portion	III	Depressed	10	Adenoma	No	HMx	VMx	Rx	63	No	Hybrid ESD	Complete	3	11	No
4	Female	75	Second portion	III	Elevated	12	Adenoma	No	Positive	Negative	R1	68	No	Hybrid ESD	Complete	4	11	No
5	Female	48	Second portion	II	Elevated	19	Adenoma	No	HMx	VMx	Rx	120	No	Hybrid ESD	Complete	9	7	No
6	Male	72	Second portion	III	Elevated	16	Adenoma	No	Positive	Negative	R1	100	ESD	Hybrid ESD	Complete	11	6	No
7	Male	74	Bulb	III	Subepithelial lesion	10	Neuroendocrine tumor	Yes	Negative	Positive	R1	43	No	Hybrid ESD	Complete	8	27	No
8	Male	63	Bulb	III	Subepithelial lesion	10	Neuroendocrine tumor	No	Negative	Positive	R1	43	No	Hybrid ESD	Complete	7	23	No
9	Female	48	Third portion	III	Depressed	21	Adenoma	No	Positive	Negative	R1	110	No	Hybrid ESD	Complete	15	9	No
10	Male	43	Bulb	III	Subepithelial lesion	8	Neuroendocrine tumor	Yes	Negative	Negative	R0	35	No	Hybrid ESD	Complete	5	15	No

*ASA*, American Society of Anesthesiologists; *ESD*, endoscopic submucosal dissection; *HMx*, involvement of the horizontal margin cannot be assessed; *R0*, curative resection with negative resection margins; *R1*, positive resection margins; *Rx*, presence of residual tumor cannot be assessed; *VMx*, involvement of the vertical margin cannot be assessed.

**Table 2:** Baseline clinicopathologic characteristics of patients in this study (n = 10)

Characteristic	n
Sex, n (%)	
Female	5 (50.0)
Male	5 (50.0)
Age, mean (SD), years	62.6 (12.4)
Location, n (%)	
Bulb	5 (50.0)
Second portion	3 (30.0)
Third portion	2 (20.0)
ASA physical status classification, n (%)	
II	1 (10.0)
III	9 (90.0)
Macroscopic type, n (%)	
Elevated (0-IIa)	3 (30.0)
Depressed (0-IIc)	2 (20.0)
Subepithelial lesion	5 (50.0)
Resection size, mean (SD), mm	12.5 (4.5)
Previous treatment, n (%)	1 (10.0)
Histological type, n (%)	
Adenoma	5 (50.0)
Neuroendocrine tumor	5 (50.0)
En bloc resection, n (%)	
Yes	4 (40.0)
Piecemeal	6 (60.0)
Resection, n (%)	
R0	2 (20.0)
R1	6 (60.0)
Rx	2 (20.0)
Horizontal margins, n (%)	
HM0	5 (50.0)
HM1	3 (30.0)
HMx	2 (20.0)
Vertical margins, n (%)	
VM0	5 (50.0)
VM1	3 (30.0)
VMx	2 (20.0)
Intraprocedural adverse events, n (%)	0 (0.0)
Postprocedural adverse events, n (%)	0 (0.0)
Rehospitalization	0 (0.0)
Anticoagulation or antiplatelet therapy	4 (40.0)
Total procedure time, mean (SD), minutes	66.5 (32.3)

0-IIa and 0-IIc refer to the Paris classification.^9^

*ASA*, American Society of Anesthesiologists; *HM0*, no involvement of the horizontal margin; *HM1*, involvement of the horizontal margin; *HMx*, involvement of the horizontal margin cannot be assessed; *R0*, curative resection with negative resection margins; *R1*, positive resection margins; *Rx*, presence of residual tumor cannot be assessed; *SD*, standard deviation; *VM0*, no involvement of the vertical margin; *VM1*, involvement of the vertical margin; *VMx*, involvement of the vertical margin cannot be assessed.

### Technical and safety outcomes

All procedures were completed by performing complete mucosal defect closure using endoscopic clips, with a mean procedure time of 66.5 (32.3) minutes. En bloc resection was achieved in 40% of the cases, with R0 resection in 20%. The lower rates were primarily attributed to submucosal fibrosis from previous interventions and technical challenges associated with neuroendocrine tumors. At the end of the resection, no residual lesions were endoscopically identified. Most importantly, no intraoperative adverse events occurred, including perforation or significant bleeding. During the follow-up period, no delayed adverse events were observed, and no patient required rehospitalization or emergency department visits related to the procedure. In 9 cases, follow-up endoscopy was performed at our institution, and no recurrence was observed. The mean follow-up period was 1007.1 (630.4) days.

## Discussion

To our knowledge, this is the first case series to report SDD after duodenal ESD in the United States. Our findings demonstrate that SDD after duodenal ESD can be safely implemented without delayed adverse events for carefully selected patients, supporting the feasibility of this approach in appropriate clinical settings. Our study builds on previous research on SDD after ESD in other parts of the gastrointestinal tract^7^^,^^8^ and extends the findings to a technically challenging duodenal location.

Complete mucosal defect closure was achieved using endoscopic clips in all cases and likely represents a favorable factor for SDD feasibility. The relatively small mean resection size (12.5 mm) may have contributed to the successful outcomes because smaller lesions are generally associated with shorter procedural times and lower adverse event rates.

However, this study has several limitations. First, the small sample size of 10 patients is insufficient to detect rare but serious delayed adverse events and limited the statistical analysis. Second, derived from a single-center experience with procedures performed by 1 highly experienced endoscopist, any generalizability of observations is limited.

Technical outcomes revealed additional challenges. The en bloc and R0 resection rates (40% and 20%, respectively) were low, with 9 out of 10 cases requiring conversion to hybrid ESD. These outcomes reflect case complexity at our tertiary referral center, including 50% neuroendocrine tumors and previous interventions that cause submucosal fibrosis, making standard ESD technically challenging. In all 10 cases, biopsies were performed before ESD, which may have contributed to the development of fibrosis. Although endoscopic full-thickness resection is also used at our institution, ESD is preferred for duodenal bulb lesions located near the pylorus, where endoscopic full-thickness resection may risk involving unaffected mucosa.

The absence of standardized discharge criteria represents another limitation. Our reliance on subjective clinical judgment limits reproducibility across different centers and endoscopists. Our follow-up methodology also had limitations, including the transfer of 1 patient to another facility after 23 days and the reliance on phone assessments for some evaluations, which may have underestimated minor adverse events.

These limitations highlight the need for larger, prospective, multicenter studies to validate our findings and establish standardized criteria for SDD after duodenal ESD. Future research should help identify specific patient and procedural characteristics that predict successful SDD, develop objective discharge algorithms, and ensure adequate safety monitoring across diverse practice settings.

In conclusion, this case series demonstrates that SDD after duodenal ESD may be feasible in appropriately selected patients with smaller lesions when performed by experienced endoscopists with complete mucosal defect closure. Although no adverse events occurred in our cohort, a small sample size and selective patient population limit broader recommendations. Standardized protocols and larger studies are essential before widespread implementation of SDD for duodenal ESD.

## PATIENT CONSENT

The requirement for written informed consent from the patients was waived due to the retrospective nature of the study.

## Disclosure

The following authors disclosed financial relationships: S. Nagao is a member of a laboratory that received research funding from AI Medical Service Inc. This affiliation had no role in the preparation of the manuscript. M. Nishimura is a consultant for Boston Scientific, Olympus America, Fujifilm, Noah Medical, and AI Medical Service Inc. M. Laszkowska and M. Nishimura received research funding from AI Medical Service, Inc. M. A. Schattner is a consultant for Boston Scientific. All authors were supported in part through the Memorial Sloan Kettering Cancer Center; National Institutes of Health/National Cancer Institute Cancer Center Support Grant (P30 CA008748). M. Laszkowska is supported by a grant from the National Institute of Diabetes and Digestive and Kidney Diseases (K08 DK125876) and the 10.13039/100000054National Cancer Institute (U01 CA265729).
